# Phytochemical Profiling and Fingerprint Analysis of Chinese Jujube (*Ziziphus jujuba* Mill.) Leaves of 66 Cultivars from Xinjiang Province

**DOI:** 10.3390/molecules24244528

**Published:** 2019-12-11

**Authors:** Lijun Song, Jie Zheng, Li Zhang, Shijuan Yan, Wenjie Huang, Jun He, Pengzhan Liu

**Affiliations:** 1 School of Food Science and Engineering & Guangdong Province Key Laboratory for Green Processing of Natural Products and Product Safety, South China University of Technology, Guangzhou, Guangdong 510641, China; slj176@163.com; 2 College of Life Science, Tarim University, Alar, Xinjiang 843300, China; cxbh1984@163.com; 3 Department of Food Science and Engineering, Jinan University, Guangzhou 510632, China; zhengjie@jnu.edu.cn; 4 Agro-Biological Gene Research Center, Guangdong Academy of Agricultural Sciences, Guangzhou, Guangdong 510640, China; shijuan@agrogene.ac.cn (S.Y.); wenjiehuang@agrogene.ac.cn (W.H.); 5 Institute of Laboratory Animal Science, Jinan University, Guangzhou 510632, China; hejun@jnu.edu.cn

**Keywords:** Chinese jujube, flavonols, leaf, phytochemical profile, polyphenols, *Ziziphus jujuba* Mill

## Abstract

Foliage of jujube (*Ziziphus jujuba* Mill.) as a byproduct of agriculture, is a traditional nutraceutical material in China. Previous studies have shown that it is a rich resource of polyphenols. However, information on its complete phenolic profile and the difference between cultivars is still limited. This study investigated and compared phytochemical profiles of leaves of 66 Chinese jujube cultivars. Forty-two compounds, including 22 flavonols, two flavanols, one flavanone, 13 derivatives of phenolic acids, three simple acids, and one unknown hexoside were identified/tentatively identified using high-performance liquid chromatography (HPLC) coupled with high-resolution mass spectrometry. Eight major flavonols were quantified by HPLC coupled with an ultraviolet (UV) detector. The contents of total flavonoids ranged from 2.6–25.1 mg/g dry weight (DW). Differences between cultivars were analyzed by hierarchical cluster analysis (HCA) and principal component analysis (PCA). This study presents a systematic study on the phenolic compounds in Chinese jujube leaves of different cultivars.

## 1. Introduction

Jujube (*Ziziphus jujuba* Mill.), belonging to the family Rhamnaceae, is widespread in Asia, Europe, and America [1]. The plant is one of the most important temperate economic crops in Eastern Asia [2], especially in China. More than four million tons of jujube fruits are harvested in China per year, which represents 90% of the total yield globally [3]. Fruit of Chinese jujube is commonly consumed as fresh, dried or as ingredients of foods due to their pleasant taste and high nutritional value; moreover, tissues such as the fruit, seed, and leaf of the plant have also been used to alleviate diseases such as palpitation, insomnia, hepatotoxicity, anemia, spleen deficiency, diarrhea, and fever in traditional medicine [4]. Results of recent studies suggest that the extracts of jujube fruits with different solvents such as hexane, ethanol or ethyl acetate are antioxidants and have potential benefits, such as anti-cancer, anti-inflammatory, hepatoprotective, and immunoregulative effects [5].

Throughout history, the jujube leaf has traditionally been used in tea drinks in China [6], it has been made into green and black tea through different processing techniques. The tea made from the young leaves of wild *Ziziphus jujuba* Mill. is commercially available and currently occupies a share in the substitute tea market in China. The product has a fresh smell and pleasant flavor. Moreover, manufacturers claim it can improve sleep, nourish the heart, and soothe the nerves [7].

The jujube leaf is documented in Chinese Materia Medica for its curative effect on children suffering from typhoid fever, furuncle, and abscess [8]. It is rich in bioactive components such as flavonoids, triterpenic acids, and saponins [7,9,10,11], and has various physiological and pharmacological functions [9,11,12]. Previous reports showed that the saponins isolated from the fresh jujube leaves have the capacity to bind and clean potential risk factors such as cholesterol from the blood [11]; the aqueous ethanol extracts of the leaves show benefits for hepatosis and wound healing in animal trials [13,14]; the extracts of jujube leaf green tea have been used to inhibit the growth of hepatocellular carcinoma cells [15].

Phenolics are second metabolites in plant tissues and have aroused the interests of researchers for their special bioactivities [16]. Several studies on the content of polyphenols in jujube leaves have been carried out [10,12,15]. The major flavonols in the leaf of wild jujube *Z. jujuba* Mill. var. spinosa (Bunge), a variety of the jujube, were quantified [7], but reports on the detailed phytochemical profiles of jujube leaves are still rare. Moreover, during thousands of years of cultivation, more than 700 subspecies, varieties, and cultivars of jujube have been developed in China and other areas. Thus, a systematical study is necessary to be conducted to reveal the inter-cultivar distribution of the main polyphenolic compounds of Chinese jujube.

For roughly 2000 years, jujubes have been widely cultivated in Xinjiang, China because the area has the most pleasant climate and soil conditions for the plant. To protect the germplasm resources, dozens of cultivars of jujube which represented the dominant ones in China have been collected and kept in the National Germplasm Resources Base of Tarim University at Alaer City, Xinjiang Province. In this study, the phytochemical profile of leaves of Chinese jujube harvested from the Base was investigated by high-performance liquid chromatography (HPLC) coupled to an ultraviolet (UV) detector and an electrospray ionization mass spectrometer (ESI-MS). Differences in contents of flavonols between 66 cultivars were analyzed using hierarchical cluster analysis (HCA) and principal component analysis (PCA). This study provided the compositional profile of leaves of different Chinese jujube cultivars without regional disparity and the results can contribute to further application of jujube leaves.

## 2. Results and Discussion

### 2.1. Identification of Phenolic Compounds

**Figure 1:**
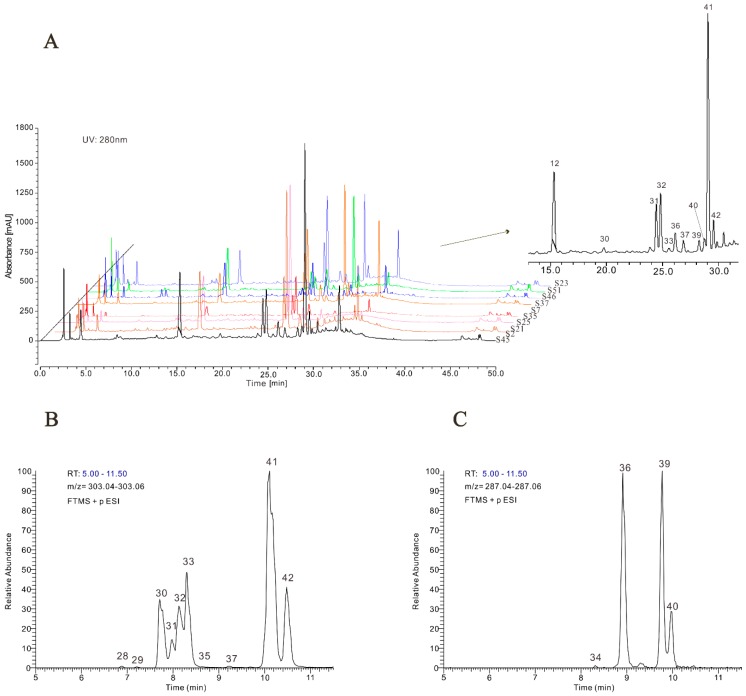
(**A**) High-performance liquid chromatography (HPLC)-ultraviolet (UV) chromatograms of extracts of jujube leaves of cultivars Mopan (S45), Chahu (S2), Tailihong (S21), Xuanchengyuan (S25), Fushuai (S35), Jikangyihao (S7), Heigeda (S37), Mayabai (S46), Xupufu (S51), and Xiaodaxiao (S23) (from the front to back) at 280 nm; (**B**) Extracted ion chromatograms (EICs) of extracts of jujube leaves of cultivar Fushuai (S35) at *m/z* 303.05; (**C**) EIC of extracts of jujube leaves of cultivar Fushuai (S35) at *m/z* 287.05. * The peak numbers were the same as Table 1.

The methanolic extracts of jujube leaves of 66 jujube cultivars were firstly analyzed with HPLC-UV. The HPLC-UV chromatograms of some typical samples recorded at 280 nm are presented in Figure 1A. The extracts of cultivar ‘Fushuai’ (S35) were selected for further analysis by HPLC-MS/MS because the chromatograms of this sample contained the most abundant peaks.

Mass spectrometry detection was performed using an HPLC system equipped with a high-resolution mass spectrometer which consisted of a linear ion trap coupled with an Orbitrap FT mass analyzer. Except for obvious peaks in total ion chromatograms (TICs), peaks in extracted ion chromatograms (EICs) of common aglycons in plant materials were chosen as candidate compounds for identification. Since the instrument can give MS and MS/MS spectra with highly accurate mass to charge ration value (*m/z*), the results were very helpful to calculate and estimate the molecular formulas of the compounds. For tentative identification, the formulas and the mass spectra of the compounds were searched against the databases of natural products such as Massbank, Metlin, National Institute of Standards and Technology (NIST), and the Kyoto Encyclopedia of Genes and Genomes (KEGG) and compared with compounds found in previous literature, especially studies on jujube species. Commercially available reference compounds were also used to verify tentative identification.

In the mass spectrometric analysis, both negative- and positive-ionization modes were tested. The negative mode was more sensitive for the acids while the flavonoids produced more fragment ions in the positive mode. Table 1 summarizes the results of identification/tentative identification, and the retention times, molecular ions, and characteristic fragment ions of each compound. A total of 42 individual compounds were identified/tentatively identified. Twenty-two glycosides of flavonols, two flavanols, a glycoside of flavanone, thirteen derivatives of phenolic acids, three simple acids and an unknown hexoside were found. For these compounds, the mass errors between the calculated molecular weight and the measured masses were less than 1.5 mmu.

#### 2.1.1. Flavonols

Glycosides of flavonols were found to be the most abundant polyphenols in the methanolic extracts of jujube leaves. A total of 22 peaks belonging to flavonols were identified and most of them were found to be the glycosides of kaempferol or quercetin. Figure 1B,C presents the extract ion chromatograms (EICs) of ions at *m/z* 303.05 and 287.05 between 5.00 to 11.00 min, which represent the typical ions fragment of quercetin moiety and kaempferol moiety, respectively. The EICs clearly showed the peaks of the glycosides of quercetin (peaks 28, 29, 30, 31, 32, 33, 35, 37, 41, and 42) and kaempferol (peaks 34, 36, 39, and 40) in the chromatograms. Peaks 15, 19, 24, 25, 27, and 38 (glycosides of quercetin) and peak 22 (glycosides of kaempferol) were also detected in the mass analyses, however, the peaks were too small to be seen. Except for derivatives of quercetin and kaempferol, a glycoside of myricetin (peak 26) was also detected.

Quercetin-*O*-glycosides can occur as monosaccharides or oligosaccharides consisting of two or more sugar units. These compounds are widely found in various fruits, vegetables, and other anatomical parts of plants [17,18,19].

Peaks 15, 19, 25, and 29 had similar mass spectra with [M + H]^+^ at *m/z* 773.21 and were tentatively identified as glycoside of quercetin for their major fragment ions at *m/z* 303.05. Two major ions of *m/z* 465.10 and 627.16 in the spectra revealed two moieties of hexose linking to the aglycon. The difference of 146.05 Da between the [M + H]^+^ and ions at 627.16 showed there was a moiety of rhamnose. Thus, these four compounds were identified as isomers of quercetin-*O*-(*di*-hexose)-rhamnose.

Peaks 24, 28, 35, and 38 were identified as glycosides of quercetin which contains a hexose, a pentose, and a rhamnose in the molecule. All of them had [M + H]^+^ at *m/z* 743.20, however, the mass spectrum of peak 35 was different from others which suggests that the linkage between the sugar units was different.

Peak 31 can be identified as rutin (quercetin-3-*O*-rutinoside) unambiguously. The compound displayed maximum absorption at 280 and 360 nm in UV and had the identical retention time with the standard compound. Three main ion fragments of *m/z* 303.05, 465.10, and 611.16 were found in its mass spectrum. Rutin was the first flavonol found in jujube leaf [9].

An isomer (peak 30) of rutin was detected, which had the same profile of UV absorption and coincident mass spectrum with rutin. However, its retention time was different from that of rutin. Quercetin-3-*O*-robinobioside (quercetin-3-rhamnosyl-(1→6)-galactoside) was detected in extracts of wild jujube (*Z*. *jujuba* Mill. var. *spinosa* (Bunge)) leaf [7], which had [M − H]^−^ at *m*/*z* 609.15, and a similar mass spectrum as peak 30 in this study. Ions at *m/z* 303.05, 465.10, and 611.16 were also found in the mass spectrum of peak 30 in positive mode. It was obvious that there were hexose and a rhamnose moieties in the structure [20]. Thus, we supposed peak 30 was quercetin-3-*O*-robinobioside and the tentative identification was confirmed by the standard compound.

Peaks 32 and 33 were isomers, which showed ions at *m/z* 303.05 and 465.10 in mass spectra, suggesting that they were hexosides of quercetin. Peak 32 with a retention time of 8.11 min was identified as hyperoside by a reference compound. Peak 33 could be quercetin-3-*O*-glucoside because the glucoside was commonly eluted slower than that of galactoside in a reverse phase HPLC system [20]. The results were verified by the standard compounds.

Quercetin-*O*-*di*-pentose (peak 37) at a retention time of 9.22 min was detected in jujube leaves for the first time. It showed ions at *m/z* 303.05, 435.09, and 567.16 in positive ion mode while simultaneously at *m/z* 301.05, 433.10, and 565.16 in negative ion mode in mass spectra. A difference of 132 Da between the ions (at *m/z* 567 and 435, at *m/z* 435 and 303) indicated there were pentose moieties (with a molecular weight of 150 Da). This glycoside was easier to be ignored because of its relatively low concentration and the seldom appearance in a few varieties.

Quercetin-3-*O*-arabinosyl-(1→2)-rhamnoside (peak 41) and quercetin-3-*O*-xylosyl-(1→2)- rhamnoside (peak 42) were the last flavonols eluted (at 10.11 and 10.42 min, respectively) in the HPLC-MS. The tentative identification was conducted by their mass spectra and the reference publications [7]. These two compounds had similar mass spectra with ions at *m/z* of 303.05, 449.11, and 581.15. The ions of *m/z* at 581.15 were identified as [M + H]^+^ because of the highest intensity in the spectra. The difference of 132 Da between the [M + H]^+^ and ions with *m/z* at 449.11 showed there was a pentose in the molecular, and the difference of 146 Da between ions with *m/z* at 449.11 and 303.05 revealed the existence of rhamnose. Quercetin-3-*O*-β-L-arabinosyl-(1→2)-α-L-rhamnoside and quercetin-3-*O*-β-d-xylosyl-(1→2)-α-L-rhamnoside were reported in previous studies, the mass spectra were similar to those of peaks 41 and 42 [7]. Through the comparison of the sequence of elution, we tentatively judged that peak 41 was quercetin-3-*O*-arabinosyl-(1→2)-rhamnoside and peak 42 was quercetin-3-*O*-xylosyl-(1→2)-rhamnoside.

Peaks 22, 34, 36, 39, and 40 all showed intense ions at *m/z* 287.05 in their mass spectra, suggesting that they were glycoside derivatives of kaempferol. Peak 22 was identified as kaempferol-*O*-(*di*-hexose)-rhamnose through its [M + H]^+^ of *m/z* 757.22 and the diagnostical ions at 595.17 and 449.11. Fragment ion patterns of peaks 36, 39, and 40 demonstrated, with references to previous reports, that they were kaempferol-3-*O*-robinobioside at 8.89 min, kaempferol-3-*O*-rutinoside (also called nicotiflorin) at 9.71 min, and kaempferol-3-*O*-glucoside at 9.96 min. The retention times of these compounds were also identical to the corresponding standards. These compounds were present in very small peaks in the chromatograms as compared to the glycoside of quercetin.

Peak 26 was the only glycoside of myricetin among the flavonols with a fragment ion of aglycon at *m/z* 319.02 in positive ion mode. The [M + H]^+^ of *m/z* 627.16 and fragment ion of 465.10 suggested a rhamnose and a hexose in the molecule.

#### 2.1.2. Flavanols and Flavanone

Catechin (peak 10 with retention time at 2.54 min) was identified based on the strong [M − H]^−^ signal at *m/z* 289.07 and [M + H]^+^ at 291.09, while its isomer epicatechin (peak 18) with an identical mass spectrum was detected at 4.12 min. Reference compounds were used to confirm the identification.

Peak 16 had [M − H]^−^ ion at *m/z* 595.17 and [M + H]^+^ at 597.18, which revealed a molecular weight of 596 Da and formula of C_27_H_32_O_15_. The pattern of its mass spectrum was very similar to that of apigenin-*C*-hexoside-*C*-hexoside reported before but with a difference of 2 Da for each fragment [21,22]. This suggested a structure of dihydro-apigenin-*C*-*di*-hexoside, and we supposed that the compounds could be naringenin-*C*-*di*-hexoside.

#### 2.1.3. Phenolic Acids

Thirteen phenolic acids were found through typical UV spectra and mass spectra. Peak 12 was the major one with three main fragments of *m/z* 191.06, 353.09, and 707.18 in negative mode, which represented the ions of the quinic acid moiety, [M − H]^−^ and [2M − H]^−^, respectively. The compound was identified as chlorogenic acid with the reference compound. Peak 17 had an identical mass spectrum with peak 12 and was identified as an isomer of chlorogenic acid, however, its retention time was not the same as the reference of neochlorogenic acid. Thus, peak 17 was an isomer of chlorogenic acid other than neochlorogenic acid. Chlorogenic acid has strong antioxidative activity with biological properties such as inhibiting obesity by improving lipid metabolism [23,24,25,26]. Further studies on phenolic acids will be conducted in the future.

Peak 5 was tentatively detected as gallic acid by its [M − H]^−^ at *m/z* of 169.01 and proved by the standard compound. Peaks 6 and 7 were identified as syringic acid and protocatechuic acid by their [M − H]^−^ at *m/z* of 197.05 and 153.02, respectively. The data was identical to the literature [21].

Four isomers of caffeic acid-*O*-hexoside were detected. The fragment ion at *m/z* 179.03 showed there was a moiety of caffeic acid and the [M − H]^−^ at 341.09 supposed these compounds to be hexosides of the acid (a difference of 162.05 Da) [21]. Based on the same method, the peaks 14 and 20 were tentatively identified as hexosides of ferulic acid.

#### 2.1.4. Other Compounds

Peaks 2, 3, and 4 were tentatively interpreted as malic acid, citric acid, and aconitic acid according to their mass spectra (Table 1). The standard compounds of malic acid and citric acid were used to verify the identification of peak 2 and 3. The tentative identification of peak 4 was made by searching the database of NIST.

Peak 1 was an unknown compound with [M − H]^−^ of *m/z* 377.09 and a fragment ion of *m/z* 215.33 in its mass spectrum. This revealed a hexose moiety in the molecule, but the structure of the aglycon needs to be identified in further study.

### 2.2. Quantitative Analysis of Flavonols in Jujube Leaves

**Figure 2:**
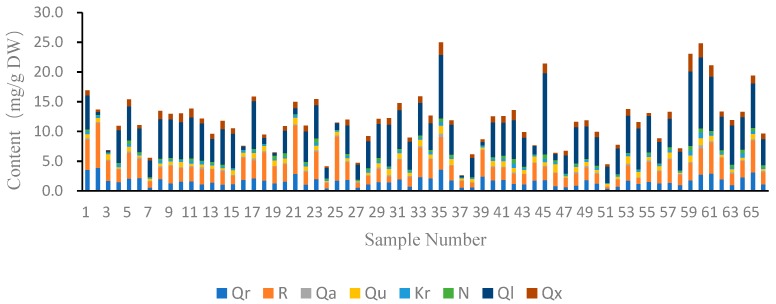
Contents of major flavonols in leaves of 66 cultivars of Chinese jujube. * The sample number and the abbreviations are as same as Table 2.

Flavonols were found to be the major compounds in the methanolic exacts of jujube leaves. The contents of eight major flavonols, including quercetin-3-*O*-robinobioside (peak 30, Qr), rutin (peak 31, R), quercetin-3-*O*-galactoside (peak 32, Qa), quercetin-3-*O*-glucoside (peak 33, Qu), kaempferol-3-*O*-robinobioside (peak 36, Kr), nicotiflorin (peak 39, N), quercetin-3*-O*-arabinosyl-(1→2)-rhamnoside (peak 41, Ql), and quercetin-3-*O*-xylosyl-(1→2)-rhamnoside (peak 42, Qx), were analyzed using HPLC-UV at 280 nm and the results are presented in Table 2. The compositional profile of 66 cultivars differed significantly (Figure 2). The contents of the other flavonols were not quantified because the peaks of them were too small to be integrated within the chromatograms.

The total flavonol contents ranged from 2.6 to 25.1 mg/g dry weight (DW) among all samples. That of the derivatives of quercetin ranged from 2.4 to 23.3 mg/g DW, while the content of quercetin-*di*-glycosides ranged from 2.0 to 21.9 mg/g DW. Extracts of Fushuai (S35), Jinsi (S60), Mopan (S45), and Long (S61) contained the highest level of flavonols in all samples with the total flavonol contents higher than 21 mg/g DW. In contrast, total flavonol contents in Xinzhengxiaoyuan (S24), Xupufuzao (S51), and Yongchengchanghong (S27) were less than 5 mg/g DW (4.1, 4.5, and 4.8 mg/g DW, respectively). Heigeda (S37) contained the lowest total flavonol content with the value less than 3 mg/g DW, while a very low level of hyperoside (quercetin-3-*O*-galactoside, Qa), quercetin-3-*O*-arabinosyl-rhamnoside (Ql), and quercetin-3-*O*-xylosyl-(1→2)-rhamnoside (Qx) were detected in this cultivar.

Quercetin-3-*O*-arabinosyl-rhamnoside (Ql) and rutin (R) were found to be the most abundant flavonols in the jujube leaves. Quercetin-3-*O*-arabinosyl-rhamnoside (Ql) was the dominated flavonol in 55 cultivars, whereas the other 11 samples like Heigeda (S37), Dadonglingzao (S3), Shanxi’niu (S19), and Chahu (S2) had a higher level of rutin (R) than quercetin-3*-O*-arabinosyl-rhamnoside (Ql). Results also indicated significant variation in concentrations of quercetin-3-*O*-arabinosyl-rhamnoside (Ql) among the cultivars. Particularly, Mopan (S45) was composed of approximately 50 times more of the compound as that of Heigeda (S37), and about 30 times as that of Shanxi’niu (S19). The total flavonol contents of Popo (S17) and Tailihong (S21) were similar, but the quercetin-3*-O*-arabinosyl-rhamnoside (Ql) content of Popo (S17) was eight times as much as Tailihong (S21), while the rutin content of Tailihong (S21) was 2.5 times as much as Popo (S17). The same situation also occurred in Chahu (S2) and Jing 39 (S11), and Xuanchengyuanzao (S25) and Xiangfen (S22). Chahu (S2) was the cultivar with the highest level of quercetin-3-*O*-robinobioside (Qr) and the second-highest level of rutin (R) while contained very little quercetin-3*-O*-arabinosyl-rhamnoside (Ql).

The contents of quercetin-3-*O*-galactoside, quercetin-3-*O*-glucoside (Qu), kaempferol-3-*O*- robinobioside (Kr), and kaempferol-3-*O*-rutinoside (nicotiflorin, N) were generally low in all the samples, ranging between 0–0.7 mg/g DW, 0–1.4 mg/g DW, 0–1.0 mg/g DW, and 0–0.8 mg/g DW, respectively.

In those samples which had less total flavonols contents, normally, there were several flavonols that could not be detected. For example, two cultivars such as Jinlingyuanzao (S39) and Naitouzao (S47) were absent of quercetin-3-*O*-glucoside (Qu), two cultivars such as Guanyin (S4) and Jiuqingfu (S40) were absent of quercetin-3-*O*-galactoside (Qa), and two cultivars named Jikangyihao (S7) and Yongchengchanghong (S27) were absent of kaempferol-3-*O*-robinobioside (Kr).

Results showed that the total flavonol contents of Chinese jujube leaves analyzed in this study could be as high as 25 mg/g. Additionally, there were two flavonols, namely quercetin-3-*O*-arabinosyl-rhamnoside (Ql) and quercetin-3-*O*-xylosyl-rhamnoside (Qx) which were not commonly found in plants. In our study, the content of quercetin-3-*O*-arabinosyl-rhamnoside (Ql) ranged between 0.3–13.7 mg/g, and the highest level was detected in the sample of Mopan (S45). The content of quercetin-3-*O*-xylosyl-rhamnoside (Qx) could reach 2.4 mg/g in Jinsi (S60). These two compounds with special sugar moiety may have special bioactive qualities for human beings, however, this needs to be evaluated by further study.

Chlorogenic acid was also quantified as the only phenolic acid in our study, the content could reach 2.2 mg/g DW in Xiaozi (S64) and down to 0.1 mg/g DW in Xinzhengxiaoyuan (S24).

### 2.3. Hierarchical Cluster Analysis (HCA)

**Figure 3:**
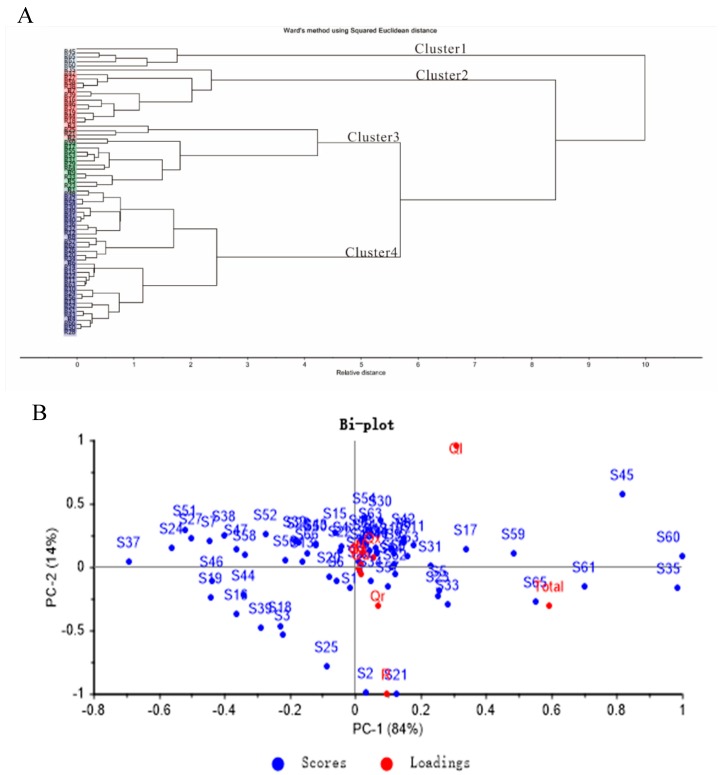
(**A**) Results of hierarchical cluster analysis (HCA); (**B**) bi-plot of principal component analysis (PCA) for contents of major flavonols in leaves of 66 cultivars of Chinese jujube. * The sample numbers are as same as Table 1 and Table 2. ** Abbreviations are as same as Figure 2.

HCA was used to categorize jujube leaf by the distribution pattern of the eight major flavonol components. The results are illustrated in Figure 3A. The samples were divided into four primary clusters and then further into several sub-clusters. Basically, the classification depended on the total contents of flavonols of the samples, but the detailed compositional profile also affected the results.

Cluster 1 consisted of five samples named Mopan (S45), Jinsi (S60), Longzao (S61), Donglingwuhe (S65), and Fushuai (S35), which were the cultivars with the top five highest total flavonol contents. However, the cluster was separated into different classes. Mopan (S45) was divided from the others first because it contained a very small amount of rutin but abundant amounts of quercetin-3-*O*-arabinosyl-rhamnoside. Meanwhile, Longzao (S61) and Donglingwuhe (S65) were separated till the end of the branch since these two cultivars had almost the same composition.

There were 12 samples in cluster 2. Among them, the total flavonol contents of five cultivars were between 4.7–7.1 mg/g DW. The high level of rutin (1.2–4.7 mg/g DW) and lower contents of quercetin-3*-O*-arabinosyl-rhamnoside (0.3–1.4 mg/g DW) could be other factors for classification; the other seven samples of Mohu (S16), Shanximutou (S18), Shanxi’niunaicui (S19), Heigeda (S37), Jinlingyuanzao (S39), Lantiandazao (S44), and Mayabai (S46) were put into this group by the software according to those components.

Cluster 3 consisted of 15 samples. The total flavonol contents of these cultivars ranged between 6.9 to 18.1 mg/g DW. Thirty-four samples were grouped in cluster 4. The total flavonol contents of most cultivars in cluster 4 were gathered around 10 mg/g DW.

### 2.4. Principal Component Analysis (PCA)

Principal component analysis (PCA) was applied to process the data matrices on flavonol contents in the jujube leaf determined by HPLC-UV. As demonstrated in Figure 3B, the PCA scatterplot with principal component 1 (PC1) and principal component 2 (PC2) showed 98% of the total variability in the flavonol data set. PC1 explained up to 84% of the total variance and was characterized mainly by contents of total flavonol, rutin, and quercetin-3-*O*-robinobioside, and the weight of the effects decreased in turns.

Similar to the HCA, the total flavonol contents of the cultivars were the predominant factor for the allocation of the samples in the PCA plot. The samples in PCA could also be divided into four groups which consisted of similar cultivars in HCA.

For PC1, the total flavonol contents of samples on the right side of the Total dot were over 20 mg/g DW, whereas on the left less than 20 mg/g DW. Obviously, the point which represents the variable Total was closely surrounded by five cultivars, Mopan (S45), Jinsi (S60), Longzao (S61), Donglingwuhe (S65), and Fushuai (S35), which was identical with samples in cluster 1 in HCA.

Tailihong (S21) was nearest to the dot of rutin because of its highest content of this compound among all cultivars, and together with cultivars which were located above that of Chahu (S2) in the PCA bi-plot composited cluster 3. The high level of rutin while lower contents of quercetin-3*-O*-arabinosyl-rhamnoside could be the main factor for the classification of these samples gathered in the PCA bi-plot. Dadongling (S3) was grouped with Chahu (S2) and Tailihong (S21) in cluster 3, but the point of S3 was on the left of the horizontal axis because of its total flavonol content was less than 10 mg/g DW. Cultivars in cluster 4 in HCA were located in the center of the PCA bi-plot.

As a statistic tool for expressing the data in such a way as to emphasize their similarities and differences, PCA has a good ability to summarize multivariate variation. The analysis could deny a limited number of principal components that describe independent variation in the results by reducing the number of dimensions. The bi-plot used in this study also provided visual information on the relation between the contents of the individual compounds and the samples.

Xinzhengxiaoyuan (S24) and Xupufuzao (S51) had similar total flavonol contents but Xupufuzao (S51) lacked quercetin-3-*O*-galactoside, so the location of S51 was higher than that of S24. In the same way, Linyilajiao (S14) with less of quercetin-3-*O*-glucoside was above Shanxibaizao (S49).

PC2 explained 14% of the difference and was contributed mainly by the contents of quercetin-3*-O*-arabinosyl-rhamnoside. Heigeda (S37) showed the farthest distance from the point of quercetin-3-O-arabinosyl-rhamnoside at the far left in the horizontal space, and indeed contained the lowest level of this compound.

The first two principal components of PC1 and PC2 were used to provide a convenient visual aid for identifying the component differences in the datasets. The PCA bi-plot was consistent with the HCA results but could provide more detailed information.

## 3. Materials and Methods

### 3.1. Plant Materials

Leaves (1.0 kg for each sample) of 66 jujube cultivars were hand-picked from the Germplasm Resources Base of Tarim University at Alaer City of Xinjiang Province, China (latitude 44°55′ N, longitude 81°28′ E) by the end of October 2017, at the same time when the mature fruits were harvested. Leaves without disease and mechanical injury and uniformed in shape were randomly chosen from each side of the tree and mixed well. After the harvesting, all samples were cleaned and dried carefully in an oven at 50 °C, and then grounded into fine powders and stored under –18 °C. All the plant materials were provided and authenticated by Song Lijun from Tarim University. The names of the cultivars are presented in Table 2.

### 3.2. Reagents

Methanol, acetic acid, and acetonitrile with HPLC gradient grade were purchased from RCI Labscan Asia Ltd. (Bangkok, Thailand).

Reference compounds (purities ≥ 98%) including malic acid, citric acid, gallic acid, chlorogenic acid (3-caffeoylquinic acid), neochlorogenic acid (5-caffeoylquinic acid), catechin and epicatechin, quercetin-3-*O*-robinobioside, rutin (quercetin-3-*O*-rutinoside), hyperoside (quercetin-3-*O*-galactoside), quercetin-3-*O*-glucoside, kaempferol-3-*O*-robinobioside, kaempferol-3-*O*-glucoside, and kaempferol-3-*O*-rutinoside, and quercetin-3-*O*-glucosyl-(1→2)-rhamnoside were purchased from Yuanye Co. Ltd. (Shanghai, China).

### 3.3. Sample Extraction

The raw materials were extracted using a common method developed in the previous study [19]. Jujube leaves powder (2.0 g for each sample) was extracted with 15 mL 70% methanol in an ultrasonic bath at 30 °C for 30 min, then the mixtures were centrifuged (2654× *g*) for 5 min and the supernatants were collected. The extraction was repeated twice, and the supernatants were combined. After that, the extracts were concentrated using a vacuum rotary evaporator to almost dry and transferred into a 10 mL volumetric flask. Pure methanol was used to bring to the scale. Finally, the samples were filtered through a 0.45 μm organic phase membrane filter and stored in sample vials (1.5 mL) under −18 °C before they were submitted to HPLC analysis. All samples were prepared in triplicate.

### 3.4. HPLC-ESI-MS Spectrometry

A Dionex Ultimate 3000 HPLC system equipped with an Orbitrap Fusion Tribrid high-resolution mass spectrometer (Thermo Fisher Scientific, San Jose, CA, USA) operating in heated electrospray ionization (ESI) mode was employed for the qualitative analysis. The parameters of mass spectrometer and HPLC followed the same method used before [27]. The instrument was controlled by Thermo Xcalibur 2.2. A reversed-phase Kinetex C18 column (100 × 2.1 mm, 2.6 μm, Phenomenex Corporation, Torrance, CA, USA) was employed and the mobile phases consisted of 1% formic acid (A) and acetonitrile (B). The gradient elution was: 0–20 min, 95–70% A; 20–30 min, 70–10% A; 30–35 min, 10% A; 35–36 min, 10–95% A; and 36–40 min, 95% A. A total flow rate of 0.5 mL/min was used and the injection volume was 10 μL.

Mass spectra in both positive and negative ion modes were recorded with full scan function in the range of m/z 100–1000. The spray voltage was 3.5 kV in positive and 3.0 kV in negative ion mode. Temperatures of ion transfer tube and vaporizer were set as 320 °C and 350 °C, respectively. The molecular ions determined in the full scan were fragmented by tandem mass spectrometry (MS/MS) via higher-energy C-trap dissociation (HCD) with a collision energy of 40 eV. The scan ranges were from *m/z* 60 to the *m/z* values of corresponding parent ions.

### 3.5. HPLC-UV

A Thermo Fisher UltiMate 3000 HPLC system coupled with a dual-wavelength UV detector (Thermo Fisher Scientific, San Jose, CA, USA) was utilized for the quantitative analysis of phenolic compounds in the extracts of samples. The method followed the previous study with modification [27]. The separation was achieved by a reversed-phase Eclipse XDB-C18 column (250 × 4.6 mm, 5 μm, Agilent Corporation, Santa Clara, CA, USA). The mobile phase consisted of acetonitrile (A) and 1% formic acid (B) and the gradient elution was: 0–25 min, 5–20% A; 25–40 min, 20–50% A; 40–45 min, 50–90% A; and 45–50 min, 90–95% A. The total flow rate was 1.0 mL/min and the injection volume was 10 μL. The temperature of the column oven was maintained at 25 °C. Chromatograms at 280 and 360 nm were acquired. The analysis was operated with a Chameleon 7.1 system manager.

### 3.6. Qualitative and Quantitative Analyses

The compounds in the methanolic extracts of jujube leaves were identified by their retention time in the chromatograms and the mass spectra in the HPLC-ESI-MS/MS. The quantification was conducted by HPLC-UV analysis after identification. Standard solutions of chlorogenic acid, quercetin-3-*O*-robinobioside, rutin (quercetin-3-*O*-rutinoside), hyperoside (quercetin-3-*O*-galactoside), quercetin-3-*O*-glucoside, kaempferol-3-*O*-robinobioside, kaempferol-3-*O*-rutinoside, kaempferol-3-*O*-glucoside, and quercetin-3-*O*-glucosyl-(1→2)-rhamnoside were prepared by resolving in methanol. Five different concentrations of each compound in the range of 0.1–2 mg/mL were prepared and analyzed by HPLC-UV. The calibration curves were constructed by plotting the peak areas versus the concentrations and used for quantification. Retention times and regression equations of ten standards in the chromatograms are shown in Table S1. The equations were used for the quantification of corresponding compounds. Quercetin-3-*O*-arabinosyl-(1→2)-rhamnoside and quercetin-3-*O*-xylosyl-(1→2)-rhamnoside were quantified relatively by calibration curves of quercetin-3-*O*-glucosyl-(1→2)-rhamnoside because of the lack of commercially available reference compounds.

### 3.7. Data Analysis

All data were collected in triplicate. Statistics analysis was performed using Microsoft Excel 2010 first. To evaluate the difference between the cultivars, hierarchical clustering analysis (HCA) and principal component analysis (PCA) were carried out by Unscrambler X software (CAMO Software AS, Oslo, Norwegian). The contents of eight principal glycosides of flavonols in jujube leaves were selected as the clustering variables.

## 4. Conclusions

This study identified/tentatively identified 42 compounds, including 22 flavonols, two flavanols, one flavanone, 13 derivatives of phenolic acids, three simple acids and an unknown hexoside in the methanolic extract of jujube leaves. The difference in phenolics composition of the leaves of 66 cultivars of Chinese jujube was investigated. The results can be utilized to help us understand the health effects of the Chinese jujube leaves and their extracts. In addition, since all the samples used in this study were grown in the same situation, the present data indicated that the contents of flavonols in jujube leaves vary with genotype. The analysis could also be used for the authentication of origins.

Due to their high content of total flavonols, *Z. jujuba* cv. Mopan, Jinsi, Longzao, Donglingwuhe, and Fushuai, may contribute to reducing the risk of heart disease, cancer, arthritis, and the aging process. In particular, Mopan contained as much as 13.66 mg/g of quercetin-3-*O*-arabinosyl-rhamnoside, and Jinsi had the highest content (2.38 mg/g) of quercetin-3-*O*-xylosyl-rhamnoside among all the samples, which might provide special bioactivities. However, further study needs to be conducted to verify such a hypothesis. In summary, Chinese jujube leaf may be exploited for the development of various functional foods and the difference in the compositional profile of cultivars may lead to their different applications.

## Figures and Tables

**Table 1:** Compounds identified/tentatively identified from leaves of Chinese jujube (*Ziziphus jujuba* Mill.).

Identification/Tentative Identification ^a^						Positive Ion Mode		Negative Ion Mode	
		RT_1_^b^	RT_2_^c^	Formula	Δ mmu	[M − H]^−^	MS^2^	[M + H]^+^	MS^2^
		(min)	(min)			(*m/z*)	(*m/z*)	(*m/z*)	(*m/z*)
1	Unknown hexoside	0.46		C_18_H_18_O_9_	0.62	377.0859	215.0328		
2	Malic acid *	0.50		C_4_H_6_O_5_	0.80	133.0139			
3	Citric acid *	0.57		C_6_H_8_O_7_	1.13	191.0197			
4	Aconitic acid	0.59		C_6_H_6_O_6_	0.97	173.0090			
5	Gallic acid *	0.82		C_7_H_6_O_5_	0.99	169.0141			
6	Syringic acid	1.03		C_9_H_10_O_5_	1.27	197.0457			
7	Protocatechuic acid	1.27		C_7_H_6_O_4_	1.03	153.0193			
8	Caffeic acid-*O*-hexoside	1.81		C_15_H_18_O_9_	1.24	341.0882	179.0348		
9	Caffeic acid-*O*-hexoside	2.17		C_15_H_18_O_9_	1.24	341.0882	179.0348		
10	Catechin *	2.54		C_15_H_14_O_6_	0.09	289.0721		291.0864	
11	Caffeic acid-*O*-hexoside	2.60		C_15_H_18_O_9_	1.24	341.0882	179.0348		
12	Chlorogenic acid *	2.98	15.08	C_16_H_18_O_9_	1.52	353.0883	191.0560		
13	Caffeic acid-*O*-hexoside	3.59		C_15_H_18_O_9_	1.24	341.0882	179.0348		
14	Ferulic acid-*O*-hexoside	3.79		C_16_H_18_O_9_	1.31	355.1038	193.0506		
15	Quercetin-(*di*-hexose)-rhamnose	3.91		C_33_H_40_O_21_	0.50			773.2140	627.1553; 465.1028; 303.0495
16	Naringenin-*C*-*di*-hexoside	4.04		C_27_H_32_O_15_	0.43	595.1680	505.1356; 385.0935; 355.0829	597.1814	
17	Chlorogenic acid isomer	4.09		C_16_H_18_O_9_	1.52	353.0883	191.0560		
18	Epicatechin *	4.12		C_15_H_14_O_6_	0.09	289.0721		291.0864	
19	Quercetin-*O*-(*di*-hexose)-rhamnose	4.29		C_33_H_40_O_21_	0.50			773.2140	627.1553; 465.1028; 303.0495
20	Ferulic acid-*O*-hexoside	4.33		C_16_H_18_O_9_	1.46	355.1039	193.0506		
21	Coumaroylqunic acid	4.59		C_16_H_17_O_8_	0.10	337.0933	191.0561	339.1075	147.0442
22	Kaempferol-*O*-(*di*-hexose)-rhamnose	5.45		C_33_H_40_O_20_	1.20			757.2197	595.1664; 449.1094; 287.0549
23	Coumaroylqunic acid	5.69		C_16_H_17_O_8_	0.10	337.0933	191.0561	339.1075	147.0442
24	Quercetin-*O*-hexose-rhamnose-pentose	5.75		C_32_H_38_O_20_	0.70			743.2036	611.1594; 465.1030; 303.0498
25	Quercetin-*O*-(*di*-hexose)-rhamnose	6.18		C_33_H_40_O_21_	0.30			773.2138	627.1557; 465.1027; 303.0500
26	Myricetin-*O*-hexose-rhamnose	6.56		C_27_H_30_O_17_	0.06	625.1420	463.0887; 317.0226	627.1557	465.1030; 319.0447
27	Quercetin-*O*-(*di*-hexose)-rhamnose	6.59		C_33_H_40_O_21_	0.50			773.2140	627.1553; 465.1028; 303.0495
28	Quercetin-*O*-hexose-rhamnose-pentose	6.83	20.96	C_32_H_38_O_20_	0.30			743.2032	611.1611; 465.1023; 303.0499
29	Quercetin-*O*-(*di*-hexose)-rhamnose	7.36		C_33_H_40_O_21_	1.20			773.2147	627.1557; 465.1029; 303.0498
30	Quercetin-3-*O*-robinobioside *	7.71	23.93	C_27_H_30_O_16_	1.23			611.1599	465.1023; 303.0496
31	Rutin (Quercetin-3-*O*-rutinoside) *	7.94	24.38	C_27_H_30_O_16_	0.68			611.1605	465.1028; 303.0498
32	Hyperoside (Quercetin-3-*O*-galactoside) *	8.11	24.98	C_21_H_20_O_12_	0.12			465.1026	303.0460
33	Quercetin-3-*O*-glucoside *	8.33	25.32	C_21_H_20_O_12_	0.12			465.1026	303.0495
34	Kaempferol-*O*-hexose-rhamnose-pentose	8.38		C_32_H_38_O_19_	1.40			727.2094	595.1666; 449.1085; 287.0554
35	Quercetin-*O*-hexose-rhamnose-pentose	8.52		C_32_H_38_O_20_	0.30			743.2032	597.1440; 465.1028;303.0497
36	Kaempferol-3-*O*-robinobioside *	8.89	25.62	C_27_H_30_O_15_	0.20			595.1660	449.1080; 287.0549
37	Quercetin-*O*-pentose-pentose	9.22	26.13	C_25_H_26_O_15_	0.85			567.1353	435.0922; 303.0503
38	Quercetin-*O*-hexose-rhamnose-pentose	9.41		C_32_H_38_O_20_	0.10			743.2030	611.1636; 465.1028; 303.0496
39	Nicotiflorin(Kaempferol-3-*O*-rutinoside) *	9.71	27.90	C_27_H_30_O_15_	0.45			595.1660	449.1080; 287.0549
40	Kaempferol-3-*O*-glucoside *	9.96	28.23	C_21_H_20_O_11_	0.16			449.1109	287.0549
41	Quercetin-3-*O*-arabinosyl-rhamnoside	10.11	28.73	C_26_H_28_O_15_	0.05			581.1503	449.1109; 303.0469
42	Quercetin-3-*O*-xylosyl-rhamnoside	10.42	29.25	C_26_H_28_O_15_	0.53			581.1505	449.1082; 303.0500

^a^ Identification of compounds with * was verified by reference compounds.

^b^ RT_1_ was obtained in the HPLC-ESI-mass spectrometry (MS) analysis.

^c^ RT_2_ was obtained in the HPLC-UV analysis.

**Table 2:** Contents (mean ± SD mg/g dry weight (DW), *n* = 3) of eight major flavonols in leaves of 66 cultivars of Chinese jujube (*Z. jujuba* Mill.).

NO.	Cultivars	Qr	R	Qa	Qu	Kr	N	Ql	Qx	T
S1	Binglang	3.53 ± 0.57	5.05 ± 0.56	0.28 ± 0.11	0.68 ± 0.03	0.43 ± 0.03	0.35 ± 0.05	5.71 ± 0.55	0.86 ± 0.01	16.90 ± 1.83
S2	Chahu	3.88 ± 0.38	7.54 ± 0.80	0.16 ± 0.01	0.70 ± 0.11	0.31 ± 0.03	0.20 ± 0.10	0.48 ± 0.19	0.39 ± 0.07	13.66 ± 1.48
S3	Dadongling	1.70 ± 0.05	3.41 ± 0.06	0.16 ± 0.01	0.89 ± 0.05	0.20 ± 0.00	0.10 ± 0.06	0.28 ± 0.00	0.11 ± 0.00	6.86 ± 0.23
S4	Guanyin	1.49 ± 0.16	2.11 ± 0.21	0.00 ± 0.00	0.27 ± 0.02	0.39 ± 0.04	0.30 ± 0.03	5.52 ± 0.49	0.69 ± 0.10	10.84 ± 1.11
S5	Haba	2.07 ± 0.19	4.32 ± 0.37	0.23 ± 0.02	0.82 ± 0.06	0.47 ± 0.04	0.56 ± 0.05	5.73 ± 0.20	1.19 ± 0.18	15.39 ± 0.71
S6	Hengyangzhenzhu	2.16 ± 0.01	3.24 ± 0.03	0.25 ± 0.01	0.30 ± 0.00	0.20 ± 0.00	0.34 ± 0.00	4.01 ± 0.02	0.54 ± 0.04	11.06 ± 0.11
S7	Jikangyihao	0.55 ± 0.01	1.05 ± 0.05	0.09 ± 0.01	0.40 ± 0.04	0.00 ± 0.01	0.11 ± 0.00	2.82 ± 0.27	0.43 ± 0.07	5.55 ± 0.20
S8	Jinchangyihao	1.96 ± 0.11	2.01 ± 0.09	0.20 ± 0.00	0.45 ± 0.02	0.51 ± 0.04	0.29 ± 0.05	6.66 ± 0.22	1.36 ± 0.41	13.43 ± 0.95
S9	Jinmangguo	1.25 ± 0.12	2.97 ± 0.27	0.17 ± 0.01	0.57 ± 0.06	0.28 ± 0.02	0.30 ± 0.02	6.46 ± 0.61	0.95 ± 0.09	12.96 ± 1.21
S10	Jinzan	1.57 ± 0.31	2.26 ± 0.38	0.19 ± 0.05	0.87 ± 0.12	0.29 ± 0.04	0.18 ± 0.04	6.19 ± 0.92	1.47 ± 0.48	13.02 ± 2.33
S11	Jing39	1.61 ± 0.34	2.40 ± 0.47	0.21 ± 0.10	0.39 ± 0.08	0.43 ± 0.21	0.45 ± 0.08	6.86 ± 1.48	1.48 ± 0.56	13.83 ± 3.11
S12	Lajiaozao	1.09 ± 0.00	2.59 ± 0.01	0.31 ± 0.14	0.49 ± 0.03	0.46 ± 0.21	0.16 ± 0.01	6.27 ± 0.19	0.80 ± 0.02	12.16 ± 0.18
S13	Lejinsanhao	1.38 ± 0.08	2.27 ± 0.12	0.10 ± 0.01	0.55 ± 0.06	0.3 ± 0.00	0.26 ± 0.02	3.88 ± 0.25	0.81 ± 0.21	9.56 ± 0.75
S14	Linyilajiao	1.05 ± 0.03	2.26 ± 0.06	0.25 ± 0.10	0.27 ± 0.11	0.25 ± 0.06	0.36 ± 0.07	5.95 ± 0.35	1.38 ± 0.34	11.77 ± 0.91
S15	Linxianyazao	1.16 ± 0.00	1.43 ± 0.03	0.11 ± 0.01	0.82 ± 0.03	0.23 ± 0.01	0.11 ± 0.00	5.78 ± 0.06	0.88 ± 0.01	10.51 ± 0.13
S16	Mohu	1.87 ± 0.02	3.74 ± 0.05	0.14 ± 0.02	0.69 ± 0.02	0.4 ± 0.00	0.03 ± 0.00	0.6 ± 0.01	0.08 ± 0.00	7.55 ± 0.02
S17	Popo	2.12 ± 0.03	3.01 ± 0.05	0.34 ± 0.05	0.92 ± 0.17	0.36 ± 0.01	0.30 ± 0.01	8.03 ± 0.22	0.75 ± 0.01	15.83 ± 0.41
S18	Shanximutou	1.75 ± 0.32	4.71 ± 0.81	0.20 ± 0.03	0.90 ± 0.21	0.3 ± 0.04	0.03 ± 0.01	1.03 ± 0.47	0.57 ± 0.43	9.47 ± 2.32
S19	Shanxiniunaicui	1.26 ± 0.03	2.86 ± 0.08	0.12 ± 0.04	0.90 ± 0.07	0.22 ± 0.00	0.59 ± 0.32	0.44 ± 0.08	0.06 ± 0.01	6.44 ± 0.01
S20	Shengli	1.55 ± 0.07	2.95 ± 0.08	0.19 ± 0.00	0.76 ± 0.06	0.28 ± 0.01	0.61 ± 0.26	3.80 ± 0.06	0.75 ± 0.15	10.88 ± 0.18
S21	Tailihong	2.88 ± 0.13	8.13 ± 0.08	0.17 ± 0.03	1.14 ± 0.20	0.46 ± 0.07	0.12 ± 0.03	1.00 ± 0.20	1.06 ± 0.51	14.96 ± 1.18
S22	Xiangfen	1.05 ± 0.04	2.16 ± 0.06	0.18 ± 0.00	0.75 ± 0.02	0.35 ± 0.00	0.39 ± 0.06	5.11 ± 0.34	0.96 ± 0.27	10.97 ± 0.78
S23	Xiaodaxiao	1.99 ± 0.06	4.61 ± 0.21	0.25 ± 0.02	0.74 ± 0.04	0.52 ± 0.03	0.72 ± 0.03	5.57 ± 0.15	1.00 ± 0.69	15.41 ± 0.49
S24	Xinzhengxiaoyuan	0.43 ± 0.35	0.98 ± 0.76	0.07 ± 0.02	0.23 ± 0.19	0.23 ± 0.08	0.18 ± 0.14	1.77 ± 1.42	0.24 ± 0.17	4.13 ± 3.14
S25	Xuanchengyuanzao	1.72 ± 0.01	7.38 ± 0.02	0.22 ± 0.06	0.63 ± 0.07	0.24 ± 0.00	0.05 ± 0.00	1.14 ± 0.31	0.11 ± 0.06	11.49 ± 0.4
S26	Yanjiamaoyuan	1.87 ± 0.10	2.99 ± 0.15	0.15 ± 0.04	0.48 ± 0.04	0.38 ± 0.02	0.32 ± 0.01	4.81 ± 0.32	0.96 ± 0.18	11.96 ± 0.86
S27	Yongchengchanghong	0.55 ± 0.00	0.78 ± 0.00	0.17 ± 0.01	0.15 ± 0.00	0.00 ± 0.00	0.18 ± 0.00	2.51 ± 0.01	0.3 ± 0.00	4.72 ± 0.05
S28	Yujing	1.09 ± 0.03	1.44 ± 0.03	0.13 ± 0.01	0.43 ± 0.09	0.38 ± 0.00	0.35 ± 0.00	4.53 ± 0.09	0.84 ± 0.17	9.19 ± 0.18
S29	Zan3	1.47 ± 0.11	2.25 ± 0.22	0.24 ± 0.03	0.69 ± 0.14	0.36 ± 0.05	0.55 ± 0.26	5.7 ± 0.59	0.85 ± 0.23	12.11 ± 0.9
S30	Zanjing	1.44 ± 0.11	1.01 ± 0.11	0.20 ± 0.03	1.04 ± 0.07	0.31 ± 0.01	0.12 ± 0.00	6.99 ± 0.29	1.10 ± 0.10	12.22 ± 0.72
S31	Zanping	1.93 ± 0.12	3.32 ± 0.16	0.18 ± 0.03	0.91 ± 0.07	0.41 ± 0.01	0.33 ± 0.01	6.51 ± 0.32	1.16 ± 0.15	14.74 ± 0.81
S32	Zhongzaoyihao	0.74 ± 0.27	1.62 ± 0.61	0.16 ± 0.04	0.58 ± 0.15	0.27 ± 0.05	0.22 ± 0.09	4.67 ± 1.76	0.71 ± 0.24	8.96 ± 3.20
S33	Bayuezuoguo	2.33 ± 0.10	4.97 ± 0.13	0.20 ± 0.01	1.11 ± 0.10	0.36 ± 0.02	0.36 ± 0.03	5.45 ± 0.22	1.12 ± 0.23	15.89 ± 0.15
S34	Banzao	2.12 ± 0.07	3.22 ± 0.14	0.13 ± 0.03	0.66 ± 0.06	0.42 ± 0.03	0.33 ± 0.01	4.48 ± 0.56	1.27 ± 0.49	12.64 ± 1.4
S35	Fushuai	3.60 ± 0.17	5.48 ± 0.01	0.68 ± 0.13	1.36 ± 0.12	0.58 ± 0.07	0.64 ± 0.18	10.69 ± 0.2	2.08 ± 0.17	25.11 ± 0.32
S36	Minzao	1.78 ± 0.07	2.46 ± 0.06	0.12 ± 0.01	1.01 ± 0.10	0.31 ± 0.01	0.40 ± 0.11	5.07 ± 0.34	0.71 ± 0.01	11.85 ± 0.01
S37	Heigeda	0.54 ± 0.44	1.18 ± 0.92	0.04 ± 0.00	0.34 ± 0.27	0.16 ± 0.02	0.04 ± 0.03	0.27 ± 0.21	0.04 ± 0.01	2.59 ± 1.89
S38	Hameizao	0.59 ± 0.03	0.80 ± 0.01	0.09 ± 0.00	0.56 ± 0.00	0.19 ± 0.00	0.08 ± 0.01	3.23 ± 0.05	0.58 ± 0.07	6.13 ± 0.05
S39	Jinlingyuanzao	2.42 ± 0.07	4.44 ± 0.13	0.11 ± 0.02	0.00 ± 0.01	0.28 ± 0.01	0.06 ± 0.00	0.56 ± 0.07	0.45 ± 0.12	8.66 ± 0.42
S40	Jiuqingfu	1.76 ± 0.01	2.23 ± 0.04	0.00 ± 0.00	0.91 ± 0.05	0.36 ± 0.01	0.27 ± 0.01	5.82 ± 0.29	1.01 ± 0.21	12.45 ± 0.61
S41	Jinzaoyihao	1.84 ± 0.07	2.11 ± 0.07	0.11 ± 0.01	0.93 ± 0.18	0.44 ± 0.04	0.49 ± 0.16	5.56 ± 0.11	1.09 ± 0.15	12.58 ± 0.75
S42	Lingbao	1.17 ± 0.09	1.72 ± 0.12	0.25 ± 0.07	0.72 ± 0.16	0.79 ± 0.12	0.74 ± 0.20	6.48 ± 0.51	1.72 ± 0.43	13.59 ± 1.7
S43	Lejinyhihao	1.10 ± 0.00	1.71 ± 0.04	0.12 ± 0.02	0.62 ± 0.02	0.28 ± 0.01	0.28 ± 0.03	4.86 ± 0.03	0.92 ± 0.21	9.88 ± 0.21
S44	Lantiandazao	1.71 ± 0.14	2.99 ± 0.21	0.10 ± 0.04	1.01 ± 0.14	0.24 ± 0.04	0.09 ± 0.00	1.41 ± 0.49	0.09 ± 0.00	7.64 ± 1.04
S45	Mopanzao	1.80 ± 0.08	2.29 ± 0.07	0.15 ± 0.01	0.61 ± 0.12	0.54 ± 0.04	0.72 ± 0.23	13.66 ± 0.21	1.60 ± 0.23	21.38 ± 0.72
S46	Mayabai	0.82 ± 0.05	2.19 ± 0.02	0.09 ± 0.02	1.37 ± 0.11	0.33 ± 0.01	0.34 ± 0.00	1.03 ± 0.03	0.18 ± 0.04	6.37 ± 0.18
S47	Naitouzao	0.63 ± 0.03	1.51 ± 0.05	0.08 ± 0.00	0.00 ± 0.01	0.24 ± 0.01	0.17 ± 0.00	3.06 ± 0.08	0.75 ± 0.11	6.72 ± 0.31
S48	Ningyanglingzao	0.91 ± 0.02	2.03 ± 0.04	0.27 ± 0.03	0.81 ± 0.11	0.27 ± 0.00	0.32 ± 0.00	6.08 ± 0.01	0.88 ± 0.02	11.57 ± 0.18
S49	Shanxibaizao	1.81 ± 0.11	2.17 ± 0.06	0.23 ± 0.01	0.66 ± 0.09	0.35 ± 0.02	0.17 ± 0.00	5.46 ± 0.23	0.99 ± 0.11	11.83 ± 0.61
S50	Tengzhoutangzao	1.21 ± 0.03	1.68 ± 0.04	0.19 ± 0.06	0.45 ± 0.04	0.37 ± 0.06	0.34 ± 0.07	4.76 ± 0.14	0.89 ± 0.25	9.9 ± 0.69
S51	Xupufuzao	0.38 ± 0.31	0.34 ± 0.24	0.04 ± 0.00	0.41 ± 0.33	0.13 ± 0.11	0.06 ± 0.04	2.72 ± 2.2	0.39 ± 0.30	4.46 ± 3.52
S52	Xiangzao	0.72 ± 0.04	1.20 ± 0.08	0.08 ± 0.00	0.42 ± 0.03	0.26 ± 0.00	0.17 ± 0.01	4.3 ± 0.41	0.56 ± 0.04	7.71 ± 0.6
S53	Zan2	1.73 ± 0.02	2.57 ± 0.02	0.29 ± 0.00	1.28 ± 0.07	0.29 ± 0.01	0.24 ± 0.01	6.26 ± 0.07	1.1 ± 0.16	13.76 ± 0.26
S54	ZL4Chen	1.20 ± 0.09	0.98 ± 0.05	0.11 ± 0.02	0.92 ± 0.08	0.31 ± 0.01	0.12 ± 0.01	6.88 ± 0.07	1.02 ± 0.00	11.54 ± 0.34
S55	Zaoshipo	1.50 ± 0.09	3.39 ± 0.15	0.24 ± 0.01	0.60 ± 0.02	0.35 ± 0.00	0.36 ± 0.02	6.15 ± 0.52	0.49 ± 0.33	13.08 ± 0.06
S56	Zan1	1.27 ± 0.00	2.06 ± 0.00	0.14 ± 0.00	0.71 ± 0.10	0.27 ± 0.01	0.16 ± 0.00	3.6 ± 0.06	0.62 ± 0.06	8.84 ± 0.01
S57	Zhongzaosanhao	1.35 ± 0.13	3.94 ± 0.16	0.17 ± 0.00	1.01 ± 0.28	0.43 ± 0.13	0.46 ± 0.13	4.79 ± 0.21	1.14 ± 0.31	13.29 ± 1.35
S58	Baodeyouzao	0.97 ± 0.02	1.78 ± 0.04	0.11 ± 0.01	0.20 ± 0.02	0.24 ± 0.01	0.16 ± 0.00	3.1 ± 0.10	0.55 ± 0.13	7.12 ± 0.34
S59	Goutouzao	1.76 ± 0.27	2.95 ± 0.45	0.19 ± 0.03	1.08 ± 0.46	0.79 ± 0.30	0.79 ± 0.32	12.55 ± 2.13	2.94 ± 1.5	23.04 ± 5.46
S60	Jinsixiaozao	2.75 ± 0.18	4.39 ± 0.26	0.52 ± 0.21	1.18 ± 0.27	0.95 ± 0.26	0.72 ± 0.03	11.93 ± 1.89	2.38 ± 0.92	24.82 ± 4.02
S61	Longzao	2.89 ± 0.05	5.24 ± 0.08	0.23 ± 0.04	0.93 ± 0.06	0.36 ± 0.01	0.43 ± 0.09	9.12 ± 0.09	1.91 ± 0.35	21.11 ± 0.51
S62	Qiding	1.94 ± 0.30	3.65 ± 0.49	0.1 ± 0.00	0.41 ± 0.11	0.37 ± 0.04	0.35 ± 0.02	5.66 ± 0.54	0.83 ± 0.03	13.31 ± 1.52
S63	Zunyi	0.96 ± 0.04	1.91 ± 0.09	0.25 ± 0.02	0.55 ± 0.05	0.32 ± 0.02	0.42 ± 0.13	6.61 ± 0.95	0.85 ± 0	11.87 ± 1.3
S64	Xiaozi	2.26 ± 0.24	2.79 ± 0.27	0.10 ± 0.00	0.46 ± 0.02	0.72 ± 0.05	0.57 ± 0.06	5.47 ± 0.39	0.9 ± 0.05	13.28 ± 1.04
S65	Donglingwuhe	3.12 ± 0.32	5.37 ± 0.51	0.2 ± 0.03	0.89 ± 0.07	0.46 ± 0.03	0.55 ± 0.04	7.49 ± 0.68	1.28 ± 0.07	19.37 ± 1.7
S66	Bayangzao	1.12 ± 0.01	2.03 ± 0.03	0.23 ± 0.11	0.31 ± 0.02	0.28 ± 0.09	0.35 ± 0.01	4.36 ± 0.21	0.95 ± 0.26	9.63 ± 0.42

* Abbreviations: Qr: quercetin-3-*O*-robinobioside, R: rutin, Qa: quercetin-3-*O*-galactoside, Qu: quercetin-3-*O*-glucoside, Kr: kaempferol-3-*O*-robinobioside, N: nicotiflorin (kaempferol-3-*O*-rutinoside), Ql: quercetin-3*-O*-arabinosyl-(1→2)-L-rhamnoside, Qx: quercetin-3-*O*-xylosyl-(1→2)-rhamnoside.

## Supplementary Materials

The following are available online at https://www.mdpi.com/1420-3049/24/24/4528/s1, Table S1: Retention time and regression equations of standards in HPLC-UV at 280 nm.
