# Supplementary material for: Phytochemical Profiling and Fingerprint Analysis of Chinese Jujube (Ziziphus jujuba Mill.) Leaves of 66 Cultivars from Xinjiang Province
Source: Molecules. 2019 Dec 11;24(24):4528. doi: 10.3390/molecules24244528 (PMC6943635; doi:10.3390/molecules24244528)
Supplement: Supplementary file 1 [file molecules-24-04528-s001.pdf]

Table S1. Retention time and regression equations of standards in HPLC-UV at 280 nm.

No.	Compounds	Rt (min)	Regression equation	R^2
1	Chlorogenic acid	15.08	y=263.74x+7.2473	0.9982
2	Neochlorogenic acid	9.73	y=236.83x+4.2132	0.9987
3	Quercetin-3-*O*-robinobioside	24.06	y=132.36x+0.6001	0.9995
4	Rutin(quercetin-3-*O*-rutinoside)	24.47	y=127.05x-1.1834	0.9981
5	Hyperoside(quercetin-3-*O*-galactoside)	25.30	y=164.82x-1.4164	0.9982
6	Quercetin-3-*O*-β-D-glucoside	25.55	y=170.45x-0.0785	0.9998
7	Kaempferol-3-*O*-robinobioside	26.07	y=165.7x-4.5449	0.9999
8	Kaempferol-3-*O*-glucoside	28.34	y=139.6x-0.1789	0.9999
9	Kaempferol-3-*O*-rutinoside	27.91	y=220.27x+5.3132	0.9999
10	Quercetin-3-*O*-glucosyl-(1→2)-	29.15	y=84.945x-0.3955	0.9996
